# Biomimetic ROS-responsive hyaluronic acid nanoparticles loaded with methotrexate for targeted anti-atherosclerosis

**DOI:** 10.1093/rb/rbae102

**Published:** 2024-08-20

**Authors:** Bingyi Li, Mei He, Zichen Xu, Qianting Zhang, Liyuan Zhang, Shuang Zhao, Yu Cao, Nianlian Mou, Yi Wang, Guixue Wang

**Affiliations:** JinFeng Laboratory, Chongqing 401329, China; Chongqing University Cancer Hospital, Chongqing 400030, China; Key Laboratory for Biorheological Science and Technology of Ministry of Education, State and Local Joint Engineering Laboratory for Vascular Implants, Bioengineering College of Chongqing University, Chongqing 400030, China; Key Laboratory for Biorheological Science and Technology of Ministry of Education, State and Local Joint Engineering Laboratory for Vascular Implants, Bioengineering College of Chongqing University, Chongqing 400030, China; JinFeng Laboratory, Chongqing 401329, China; Key Laboratory for Biorheological Science and Technology of Ministry of Education, State and Local Joint Engineering Laboratory for Vascular Implants, Bioengineering College of Chongqing University, Chongqing 400030, China; Key Laboratory for Biorheological Science and Technology of Ministry of Education, State and Local Joint Engineering Laboratory for Vascular Implants, Bioengineering College of Chongqing University, Chongqing 400030, China; Key Laboratory for Biorheological Science and Technology of Ministry of Education, State and Local Joint Engineering Laboratory for Vascular Implants, Bioengineering College of Chongqing University, Chongqing 400030, China; Key Laboratory for Biorheological Science and Technology of Ministry of Education, State and Local Joint Engineering Laboratory for Vascular Implants, Bioengineering College of Chongqing University, Chongqing 400030, China; College of Basic Medical Sciences, Chongqing Medical University, Chongqing 400016, China; JinFeng Laboratory, Chongqing 401329, China; Key Laboratory for Biorheological Science and Technology of Ministry of Education, State and Local Joint Engineering Laboratory for Vascular Implants, Bioengineering College of Chongqing University, Chongqing 400030, China

**Keywords:** nanomaterials, atherosclerosis, ROS-responsive, methotrexate, targeted delivery

## Abstract

Atherosclerosis (AS), an inflammatory disease characterized by lipid accumulation, has a high global incidence and mortality rate. Recently, nanotherapeutic approaches that target pathological sites and improve drug bioavailability and biocompatibility hold great promise for AS treatment. In this study, a biomimetic ROS-responsive hyaluronic acid–based nanomaterial was prepared for targeted anti-AS. Specifically, a safe ROS-responsive carrier based on hyaluronic acid (HSP) was prepared to load methotrexate (MTX), a drug known for its ability to enhance lipid excretion, resulting in the formation of MTX-loaded nanoparticles (MTXNPs). Furthermore, the macrophage membrane was coated on the surface of MTXNPs to obtain MM/MTXNPs. Both MTXNPs and MM/MTXNPs exhibited ROS responsiveness and demonstrated excellent biocompatibility. *In vitro* experiments revealed that MM/MTXNPs could evade macrophage phagocytosis and exhibited high uptake rates by inflamed endothelial cells. MM/MTXNPs also reduced lipid accumulation in foam cells. *In vivo* experiments showed that MM/MTXNPs exhibited superior accumulation at AS plaque sites, facilitated by the surface membrane layer containing integrin α4β1 and CD47, resulting in an enhanced therapeutic effect in inhibiting plaque development compared to free MTX and MTXNPs. Therefore, HSP represents a promising nanocarrier to load hydrophobic MTX, enabling effective and biocompatible enhancement of AS treatment.

## Graphical abstract

**Graphical Abstract:**
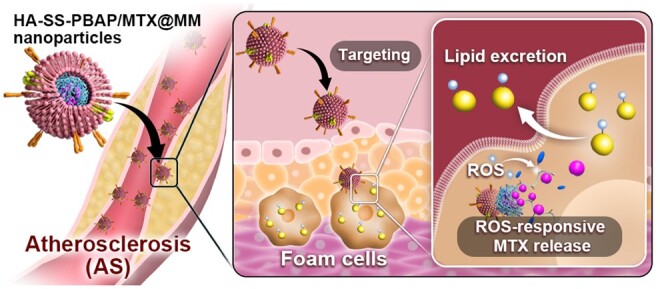

## Introduction

Cardiovascular disease is the leading cause of illness and death worldwide [1]. Atherosclerosis (AS) is thought to result from a nonadaptive inflammatory response that is triggered by the retention of cholesterol and Apo B-rich lipoproteins in the more vulnerable areas of the arterial walls [2, 3]. Oral lipid-lowering drugs are commonly prescribed for the treatment of AS. However, they can potentially lead to various adverse effects such as muscle pain, abnormal liver function and gastrointestinal discomfort [4]. In recent years, the administration of drugs through drug delivery systems has demonstrated the advantages of improving drug efficacy and reducing therapeutic side effects [5]. However, the challenge of overcoming immune barriers in drug delivery systems must be taken into consideration. In this regard, significant progress has been made in the field of biomimetic nano-strategies [6]. The advantage of using immune cells to actively deliver drugs and drug-loaded nanoparticles to targets stems from the inherent transport capabilities of living cells, such as circulation within the body, active traversal of biological barriers and chemotactic targeting towards diseased sites [7]. The specific biological functions of the source cells are inherited by the core nanoparticles through the fusion of natural cell membranes [8, 9].

Macrophages are a characteristic feature of ASCVD and perform a significant function in the formation of plaques, local inflammation and thrombosis [10]. Because of its large surface area, strong drug loading capacity, high drug stabilization potential, long cycle time and possible enhancement of accumulation and penetration in diseased tissues, drugs targeting macrophages and drug delivery strategies are emerging as a means to treat disease [11]. CD47, which is also referred to as integrin-associated protein, is a transmembrane protein. As a primary immune cell checkpoint for macrophages, CD47’s principal purpose is to resist phagocytosis or emit a ‘don’t eat me’ signal that enables cells to avoid phagocytosis and removal by macrophages and other phagocytes [12]. VCAM-1 is substantially expressed on the surface of endothelial cells at AS plaques during inflammation and can attach to integrin α4β1 [13]. Consequently, macrophage membrane (MM)-enclosed nanoparticles enable the avoidance of clearance and precise targeting of AS plaques *in vivo*.

In AS, ROS promotes endothelial dysfunction and the expression of inflammatory factors such as MCP-1, TNF-α and IL-1 [14]. These inflammatory factors, in turn, induce the production of ROS, resulting in the formation of a vicious cycle. Additionally, *in vitro*, ROS can also promote the formation of foam cells and the proliferation and migration of vascular muscle cells [15]. Based on the high ROS environment at AS lesion sites [16, 17], designing a nanoparticle that can respond to ROS may be able to provide better efficacy at AS lesion sites. ROS-responsive materials, such as sulfur-, selenium- and tellurium-based reactive polymers, phenylboronic acid polymers and oxalic acid materials, exhibit the ability to undergo hydrophilic and hydrophobic transformations, as well as degrade under high ROS levels [18–20]. The utilization of these materials in nanocarriers holds promise for delivering precise localized therapy. According to studies, 3,3′-Dithiodipropionic acid has a disulfide bond and can be used as an intermediate in response to glutathione [21]. 4-Hydroxyphenylboronic acid pinacol ester (PBAP) is selectively responsive to H_2_O_2_ and exhibits excellent *in vitro* degradation kinetics at physiologically relevant H_2_O_2_ levels [22]. The objective of this study was to develop ROS-responsive carriers by conjugating these two responsive groups to a biocompatible hyaluronic acid (HA) backbone. This innovative approach allows for self-assembly and efficient loading of hydrophobic drugs onto the core.

Studies have demonstrated that methotrexate (MTX) elevates intracellular levels of adenosine monophosphate and adenosine. The upregulation of adenosine receptors can restrict the formation of foam cells by facilitating lipoprotein efflux from macrophages [23–25]. Hence, MTX can potentially be utilized as a therapeutic agent to inhibit lipid accumulation in AS [23]. Due to its hydrophobic nature and low solubility in the physiological environment, MTX is difficult to administer efficiently. However, reformulating MTX into nanoparticles can overcome these limitations, thereby maximizing the potential therapeutic benefits of this drug. Paolo’s team developed nanoparticles that encapsulate MTX within liposomes for the treatment of AS. The team discovered that liposome-encapsulated MTX was significantly more effective at hindering AS progression in ApoE^−/−^ mice than administration of free drugs [26].

**Figure 1.**
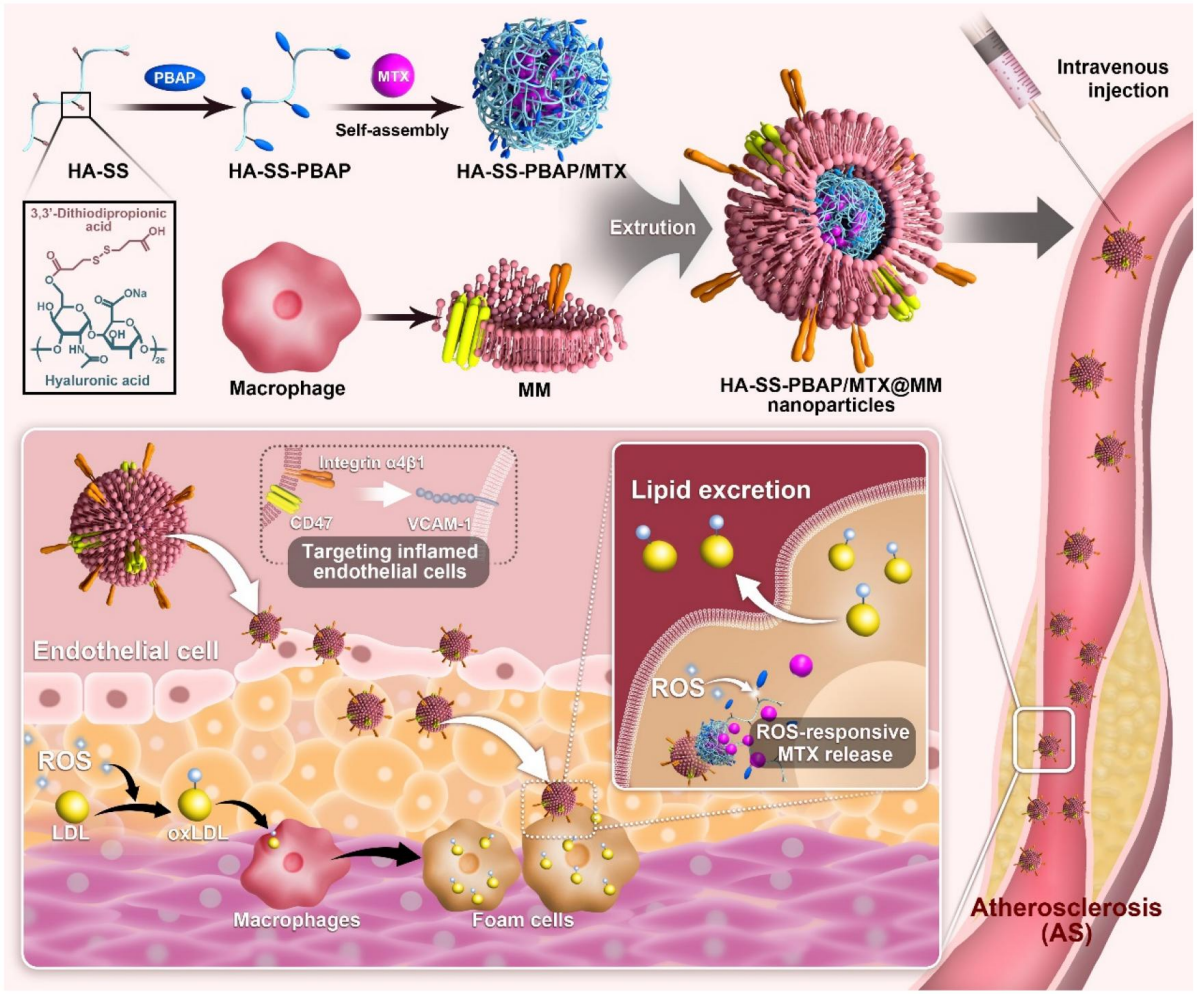
Schematic of MM/MTXNPs fabrication and its treatment for AS.

Therefore, based on the large amount of lipid deposition in AS lesions, the highly expressed ROS inflammatory environment and the high expression of adhesion molecules in endothelial cells, our study used HA, known for its high safety profile, as the backbone to tether ROS-responsive groups. This enabled the development of ROS-responsive HSP carriers, subsequently loaded with the lipid-efflux-promoting drug, MTX, to form the nanomedicine MTXNPs. Moreover, we utilized MMs to encapsulate the nanomedicines, achieving a ‘stealth’ effect while specifically targeting inflammatory endothelial cells at the AS lesion site. This targeted delivery facilitated drug release at the plaque site, effectively impeding the progression of AS (Figure 1).

## Materials and methods

### Materials

HA (MW 10 000), 3,3′-dithiodipropionic acid, PBAP, formamide, 1-ethyl-(3-dimethylaminopropyl) carbodiimide (EDC) and 4-dimethylaminopyridine (DMAP) were all obtained from Macklin (Shanghai, China). MTX was procured from Sangon Biotech (Shanghai, China). Membrane and Cytosol Protein Extraction Kit, Kit-8 Cell Counting Kit and DAPI were purchased from Shanghai Beyotime Biotechnology. DID was purchased from Aladdin (Shanghai, China). An Oil Red O staining kit (for cultured cells) was purchased from Solarbio (Beijing, China).

### Synthesis of HSP

In this process, 0.5 mmol HA is first dissolved in 20 ml of formamide. Then, 3.9 mmol of EDC and 3.9 mmol of DMAP were added slowly to the solution. The mixture was then heated in an oil bath at a constant temperature of 40°C for 4 h. Then, 6.5 mmol of 3,3′-dithiodipropionic acid was added to the flask, and the reaction was continued at 40°C overnight. The reaction solution was subsequently subjected to dialysis to eliminate any impurities. To activate the hydroxyl groups of PBAP, 3.9 mmol of PBAP, along with 4.68 mmol of EDC and 4.68 mmol of DMAP, were dissolved in 5 ml of DMSO and heated in an oil bath at 40°C for 4 h. Afterward, 0.3 mmol of the reaction product from the previous step was dissolved and added to the activated PBAP solution, and the reaction mixture was kept in an oil bath at 40°C overnight. This step allowed for further chemical modification of the PBAP polymer for subsequent use in various applications. Once the reaction was complete, the final product solution was then purified by dialysis to remove impurities. Then, the resulting product solution was collected and subjected to lyophilization to obtain a light brown solid. This solid is known as the HSP carrier, which can be used for further chemical modifications or various applications.

### Preparation of the MMs

The extraction of MMs was conducted by established methods [8], with minor modifications, as detailed below. The collected macrophage suspension was placed into a 2-ml centrifuge tube and spun at 1500 rpm for 10 min at a temperature of 4°C. Once this was completed, the supernatant was removed and Cell Extraction Reagent A with PMSF was added to the tube containing the cell precipitate. The mixture was then placed on ice for 15 min. Next, the cells underwent three freeze–thaw cycles in liquid nitrogen, and they were subsequently centrifuged at 700 × g for 10 min at a temperature of 4°C. The supernatant was then transferred to a new centrifuge tube, and further low-temperature (4°C) centrifugation was conducted at 14 000 × g for 30 min to discard any remaining supernatant and acquire the desired precipitate, which was the MM. The membrane was further resuspended using ultrapure water and eventually stored at −80°C.

### Preparation of MTX-loaded HA nanoparticles

We used a nanoprecipitation method to prepare MTX-loaded HA nanoparticles (MTXNPs). First, 10 mg of HSP and 1 mg of MTX were accurately weighed and dissolved in 1 ml of formamide before they were added to 4 ml of ultrapure water with vigorous stirring. The resulting solution was then transferred to a dialysis bag with a 3500-Da cutoff for 12 h. During this time, the water was changed every 4 h to eliminate the organic solvent and any unencapsulated MTX.

DiDNPs were produced by co-dissolving 10 mg of HSP and 1 mg of MTX in formamide, followed by the dropwise addition of DiD, under light-excluded conditions. The 1-ml organic phase mixture was then dialyzed dropwise into 4 ml of the aqueous phase for approximately 5–6 h.

### Preparation of MM camouflaged MTXNPs

MTXNPs were mixed with MM at a ratio of 9–1 by volume and subsequently sonicated in a water bath at 42 kHz and 100 W for a duration of 8 min. The resulting mixture of nanoparticles and MMs was then transferred into a liposome extruder, with the final product being collected as MM camouflaged MTXNPs (MM/MTXNPs) after 14 rounds of extrusion, each with a pore size of 400 nm.

### Characterization of the nanoparticles

^1^H-NMRThe final HSP product was dissolved in deuterated water. This sample was then subjected to ^1^H-NMR analysis using a Bruker Avance NEO instrument from Germany, featuring a magnetic field strength of 400 MHz.

FTIR: HA, 3,3′-dithiodipropionic acid, PBAP, HA and HA linkages, as well as the final HSP product, were precisely weighed and then characterized using Fourier transform infrared spectroscopy. Thermo Scientific Nicolet 6700 from the USA was employed for this analysis.

Size and Zeta potential: The particle size and zeta potential of MTXNPs, MM/MTXNPs and cell membrane vesicles were assessed at room temperature using a Malvern Zetasizer Nano ZS unit (Nano ZS 90/Malvern, U.K.), with three parallel measurements obtained for each sample.

Transmission electron microscopy (TEM): The TEM morphology of MTXNPs and MM/MTXNPs was investigated via TEM. The nano solution with a concentration of 100 μg/ml was introduced dropwise onto a copper grid before being negatively stained using phosphotungstic acid. The samples were then allowed to dry before being observed on the machine.

### Characterization of cell membrane proteins

Membrane proteins were determined through the use of polyacrylamide gel electrophoresis (SDS–PAGE). Total macrophage proteins, MMs and MM/MTXNPs were prepared following the procedures described previously by us/researchers. The protein samples were lysed using cell lysis solution and then collected for analysis. A 10% Bis–Tris gel was prepared according to the instructions of the kit, and the samples were added to the gels. The Bio-Rad electrophoresis system was run at a constant voltage of 60 V for 50 min. The gels were then stained with SimplyBlue to visualize the results.

The expression of CD47 and integrin α4β1 in RAW264.7 cells, MMs and MM/MTXNPs was confirmed by western blotting. Eight percent SDS–PAGE gels were prepared by following the instructions provided in the kit, and protein samples for total macrophage proteins, MM and MM/MTXNPs were prepared according to the protein extraction kit instructions. Electrophoresis was performed using SDS–PAGE, followed by transfer to polyvinylidene difluoride membranes and incubation with CD47 (anti-CD47 antibody, WH274452, Abclonal), α4 (anti-integrin α4, 8440 T, CST) and β1 (anti-integrin β1, ab16048, Abcam) primary antibodies followed by treatment with HRP-labeled goat anti-mouse IgG (H + L) (Beyotime, Jiangsu, China). Protein signals were measured using the ChemiDoc MP imaging system (Bio-Rad, USA) through an enhanced chemiluminescence method.

### Drug release

The MTXNPs were freeze-dried, weighed and re-dissolved in formamide solvent. The absorbance values at 302 nm were measured using a UV/Vis spectrophotometer (DU730, Beckman Coulter), and these data were used along with a standard curve to determine the corresponding drug content. The drug loading efficiency (LE) and drug encapsulation efficiency (EE) were calculated as follows:

$$\begin{array}{c} LE（\%）=\frac{M_{\mathrm{MTX}}}{M_{\mathrm{HSP}}+M_{\mathrm{MTX}}}\times 100\%\\ EE（\%）=\frac{M_{\mathrm{MTX}}}{M_{\mathrm{added}}}\times 100\% \end{array}$$

*M*_MTX_: the amount of MTX loaded in the NPs.

*M*_HSP_: the amount of polymer in the formulation.

*M*_added_: The quantity of added MTX.

Separate nano solutions were prepared for MTXNPs and MM/MTXNPs (2 mg/ml, 1 ml) using 3500 Da dialysis bags as per previously described methods. The release media for drug release measurements were water, aqueous solutions of 1 mM H_2_O_2_, 1 mM GSH and a combination of 1 mM H_2_O_2_+1 mM GSH. The experiments were conducted in triplicate, and drug release was measured using a constant temperature shaker at 37°C. The release solution was collected and aspirated at specific time intervals of 0, 1, 2, 4, 8, 12, 24, 48, 72, 96, 120 and 144 h. The collected solution was then replenished with an equal volume of fresh release medium, and the absorbance values were measured at 302 nm using a UV/Vis spectrophotometer (DU730, Beckman Coulter).

### Cellular uptake

Preparation of DiDNPs and MM/DiDNPs: 10 mg of HSP carrier and 1 mg of MTX were co-dissolved in formamide, and DiD was added dropwise to the solution. This process was carried out under light-proof conditions. Next, 1 ml of this organic phase was carefully added dropwise to 4 ml of an aqueous phase under light-proof conditions. DiDNPs were obtained by dialyzing the mixture for 5–6 h. MM/DiDNPs were obtained by co-extruding the DiDNPs with MMs using a specific method/apparatus.

Experiments on RAW264.7 phagocytosis: RAW264.7 cells were seeded at a density of 5 × 10^5^ cells/well and incubated overnight. Next, 100 μg of DiDNPs and MM/DiDNPs (obtained by dilution in culture medium) were added to each well. The cells were then incubated with DiDNPs for 0.5, 1, 2 and 4 h. Subsequently, the cells were stained with DAPI and observed under laser confocal microscopy before being photographed.

Experiments on HUVEC phagocytosis: HUVECs were cultured at a density of 5 × 10^5^ cells per well. Inflammatory HUVECs were induced by treating the cells with 1 µg/ml LPS for 12 h. Next, 100 μg of DiDNPs and MM/DiDNPs were added to the wells of both HUVECs and inflammatory HUVECs. Both DiDNPs and MM/DiDNPs were diluted in culture medium. The cells were then incubated for a total of 4 h. The nuclei were stained with DAPI, and the cells were observed using laser confocal microscopy and photographed.

### Foam cell induction

To induce foam cell formation, RAW264.7 cells were seeded at a density of 1 × 10^6^ cells and incubated for 6 h. The complete medium was then removed, and the cells were subjected to starvation treatment for another 8 h. Next, the cells were treated with oxLDL at concentrations of 0, 20, 50, 80 and 100 μg/ml and incubated for 24 h. Foam cells were detected by staining with oil red O.

### Promoting lipid excretion

Inhibition of macrophage lipid phagocytosis: RAW264.7 cells were co-incubated with oxLDL at a concentration of 50 μg/ml. Three groups, consisting of MTX, MTXNPs and MM/MTXNPs, were set up to administer drugs at an effective concentration of 10 μg/ml MTX in each group. The cells were then co-incubated with oxLDL and the respective treatments/drugs for 12 h. Afterward, oil red O staining was performed for identification purposes.

Enhanced lipid excretion in foam cells: Foam cells were created by incubation with 50 μg/ml oxLDL and then treated with three different doses of MTX, MTXNPs and MM/MTXNPs at an effective concentration of 10 μg/ml MTX. The cells were then subjected to oil red O staining for identification after 12 h.

### Cell cytotoxicity evaluation

ECs and RAW264.7 cells were seeded into 96-well plates at a density of 1 × 10^4^ cells/well and incubated for 12 h. NPs were then added, followed by treatment with culture medium containing varying doses of nanoparticles or MM-coated nanoparticles (MM/NPs). After co-incubation for 8 h, cell viability was determined by using the CCK-8 assay.

### *In vitro* blood compatibility tests

One milliliter of fresh anticoagulated C57BL/6 mouse blood was mixed with 1.25 ml of saline. MTXNPs and MM/MTXNPs were diluted to a concentration of 1 mg/ml, and 1 ml of the diluted nanoparticles was added to 2 ml of the mixture along with 20 μl of the anticoagulated blood. The mixture was incubated in a 37°C water bath for 1 h, followed by centrifugation at 2000 rpm for 5 min. The absorbance of the supernatant was measured at 545 nm using a microplate reader (μQuant, Bio-Tek Instruments Inc., Winooski, USA) to determine the amount of hemoglobin released from lysed erythrocytes. Saline was used as a negative control, and triple-distilled water was used as a positive control.

### Animals

C57BL/6 male mice and apolipoprotein E knockout (ApoE^−/−^) mice at 7 weeks of age were obtained from the Changzhou Cavens Laboratory Animal Co., Ltd, China. The animals were fed according to the rules. Before the experiment, these mice underwent one week of acclimatization with adaptive feeding. All animal procedures were approved by Laboratory Animal Welfare and Ethics Committee of Chongqing University for Animal Protection (CQU-IACUC-RE-202306-001).

### *In vivo* long-term circulation test

Six mice were randomly divided into two groups. In one group, mice were injected with 200 μl of DiDNPs (at a concentration of 2 mg/ml), while in the other group, mice were injected with MM/DiDNPs (also at a concentration of 2 mg/ml). Blood samples (20 μl) were collected from the tail of each mouse at 1, 6, 12, 24 and 48 h after injection. The blood was diluted with EDTA-2K (a type of anticoagulant) and stored in a refrigerator at 4°C until all samples were collected for further testing.

### *In vivo* targeting of atherosclerotic plaques

The ApoE^−/−^ mice, which were successfully modeled, were randomly divided into three groups consisting of three mice each. Mice were injected with 200 μl of DiDNPs and MM/DiDNPs at a concentration of 2 mg/ml via the tail vein, while the control group received an injection of an equal amount of PBS. Twelve hours after injection, the mice were anesthetized and euthanized, and their hearts, livers, spleens, lungs, kidneys and blood vessels were perfused with saline containing heparin. The organs were then macerated in 4% PFA and stored in a light-protected refrigerator at 4°C until further imaging under a small animal imaging system.

### Treatment of AS in ApoE^−/−^mice

Twenty ApoE^−/−^ mice were randomly divided into four groups with five mice in each group. The mice were injected with a solution of PBS, MTX, MTXNPs and MM/MTXNPs at a concentration of 200 μg/ml via the caudal vein every 3 days at a dosage of 1.0 mg/kg. The treatment lasted for 30 days, and the mice were maintained on a high-fat diet throughout the treatment period.

### Quantitative analysis of the atherosclerotic plaques

The dissected aorta was carefully unfolded to remove fat and adherent tissue from the outer wall, after which it was dissected longitudinally and fixed in 4% paraformaldehyde in PBS. The plaque area was quantified using the oil red O staining method. To determine the extent of AS in the aortic root, the tissue was cryosectioned and then stained with ORO to quantify the area of atherosclerotic plaque using ImageJ software.

### Histology and immunohistochemistry

The aortic arch root was surgically cut and then treated with an antigen repair solution for fixation and repair purposes. After that, we conducted Masson staining and immunohistochemical analysis. To inhibit endogenous peroxidase activity, the sections were soaked in a solution of 3% hydrogen peroxide and 100% methanol for 20 min. Subsequently, the sections were treated with 1% bovine serum albumin in PBS containing 0.3% Triton X-100 for 60 min. For the quantification of macrophages and smooth muscle cells, we incubated the sections with CD68 and alpha-smooth muscle actin (α-SMA). Major organs such as the heart, liver, spleen, lung and kidney were harvested, fixed with 4% paraformaldehyde in PBS and subjected to histological analysis using hematoxylin–eosin (H&E) staining.

### Complete blood count and clinical chemistry

Once the mice were anesthetized, their blood was collected into anticoagulation tubes, which were then divided in half. One of those halves was analyzed for levels of cholesterol (TC), triglycerides (TG), glutathione aminotransferase (ALT), aspartate aminotransferase (AST), urea nitrogen (BUN) and creatinine (CREA). The other half of the whole blood was utilized to analyze blood count, which included red blood cells, white blood cells, platelets and hemoglobin level.

### Statistical analysis

The data obtained in this study are presented as the means ± SDs. The statistical analysis was carried out using GraphPad Prism Version 6.0 software (GraphPad, USA). To compare the groups, we employed one-way analysis of variance (ANOVA) with Tukey’s test. Significance levels were noted as *P* < 0.001***, *P* < 0.01** and *P* < 0.05*.

## Results

### Synthesis and characterization of MM/MTXNPs

**Figure 2.**
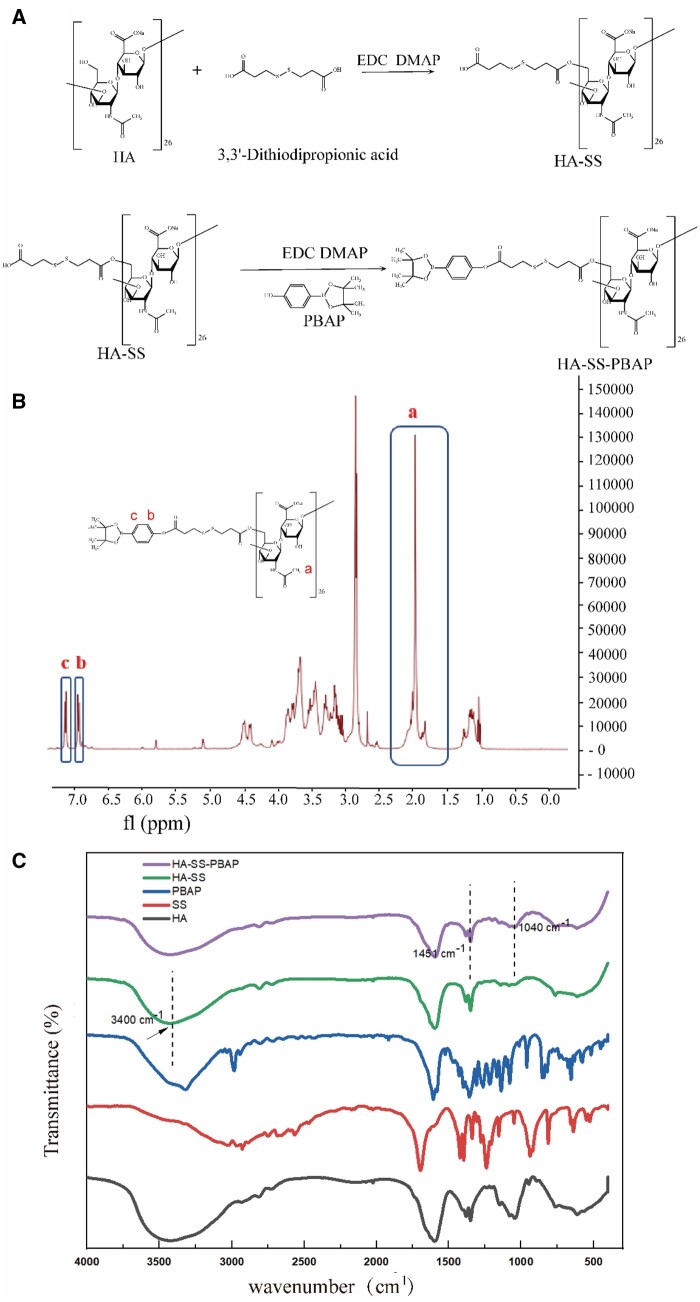
The synthesis pathway and characterization of HA-SS-PBAP. (**A**) Synthetic route of HA-SS-PBAP. (**B**) ^1^H-NMR characterization of HA-SS-PBAP in D_2_O. (**C**) Representative Fourier transform spectroscopy (FTIR) absorption spectra of HA, SS, PBAP, HA-SS and HA-SS-PBAP.

Figure 2A shows the synthetic pathway for HA-SS-PBAP. The ester bond is formed by coupling the carboxyl group of 3,3′-dithiodipropionic acid with the hydroxyl group of HA, creating the precursor HA-SS. We combined this precursor with PBAP through a reaction process to create the HA-SS-PBAP carrier. Fourier transform infrared spectroscopy (Figure 2C) shows a wide peak at 3400 cm^−1^, indicating that the hydroxyl bond of HA is linked to 3,3′-dithiodipropionic acid, essentially neutralizing the free carboxyl group. Following the coupling process with PBAP, the carboxyl peak of 3,3′-dithiodipropionic acid at 3400 cm^−1^ disappeared, while two peaks emerged at 1451 and 1440 cm^−1^ for HA-SS and HA-SS-PBAP. These peaks correspond to –NHCO–, indicating the presence of this functional group in the compounds. Moreover, HA-SS-PBAP maintained the crucial characteristic peaks of Ar, which signifies the productive synthesis of the coupling. Figure 2B shows the chemical structures of HA-SS-PBAP, which were also characterized utilizing ^1^H-NMR. HA-SS was identified by its peaks within the 1.9–2.1 ppm range, indicating a methyl single peak. The peak detected at 2.65–2.85 ppm was identified as the methylene group adjacent to the disulfide bond in HA-SS-PBAP. Additionally, the characteristic peak of HA-SS-PBAP was observed within the 6.8–7.2 ppm range, corresponding to the hydrogen located on the benzene ring of HA-SS-PBAP. This outcome implies that PBAP effectively binds to HA-SS.

**Figure 3.**
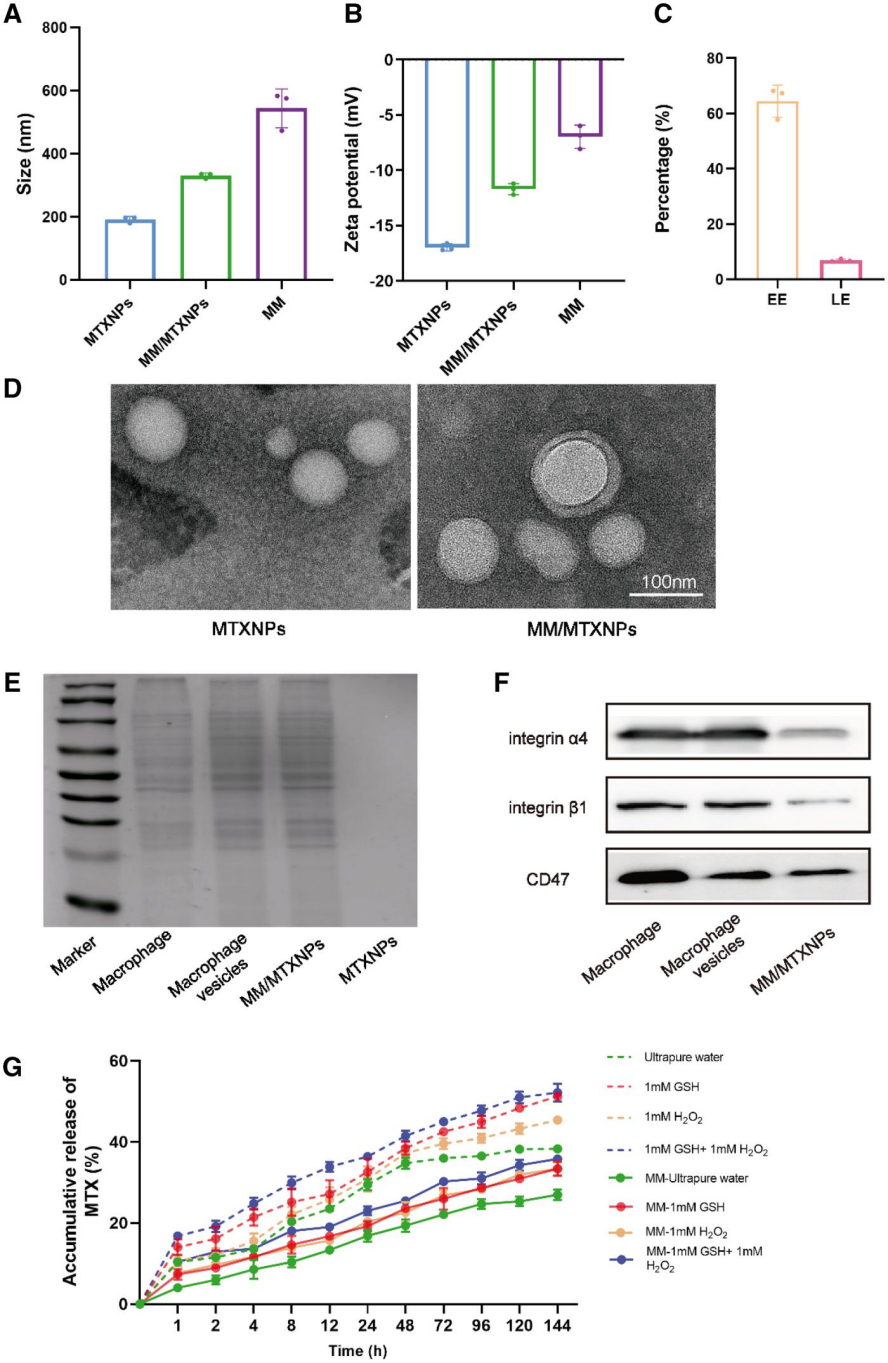
Characterization of the MM-coated biomimetic nanoparticles. Hydrated particle size (**A**) and zeta potentials (**B**) of MTXNPs, MM/MTXNPs and MM (*n* = 3). (**C**) The encapsulation and loading rates of MTXNPs (*n* = 3). (**D**) TEM images of MTXNPs and MM/MTXNPs (the scale bar is 100 nm). (**E**) SDS–PAGE images of membrane proteins of total macrophage proteins, macrophage vesicles, MM/MTXNPs and MTXNPs. (**F**) Western blot of integrin α4β1 and CD47 on macrophages, macrophage vesicles and MM/MTXNPs. (**G**) The release behavior of MTXNPs and MM/MTXNPs *in vitro* (*n* = 3).

Dynamic light scattering (DLS) analysis (Figure 3A) revealed that the MTXNPs had a particle size of 184.1 ± 2.8 nm (PDI: 0.191 ± 0.033). Furthermore, the analysis (Figure 3B) showed that the MTXNPs had a potential of −16.9 ± 0.3 mV, indicating their uniform particle size, good dispersion and negative surface charge. The MTXNPs were prepared using the nanoprecipitation method, resulting in an encapsulation rate of 65.43% and a drug loading rate of 7.89% (Figure 3C). According to the TEM images, the MTXNPs displayed spherical structures with an average diameter of 80 nm (Figure 3D). The particle size of MM/MTXNPs was 327.8 ± 7.8 nm (Figure 3A), with a potential of −11.7 ± 0.5 mV (Figure 3B). This observation indicates that the particle size was increased in comparison to that of MTXNPs. This increase was attributed to the alteration of the MM coating on the surface of the nanoparticles. From Figure 3D, it is evident that MM/MTXNPs display a distinct ‘coreshell’ configuration, where the ‘core’ is MTXNPs and the ‘shell’ is the macrophage cell membrane, approximately 10 nm in thickness. The DLS and TEM analyses yielded consistent results, indicating the successful formation of uniformly sized MTXNPs and confirming the successful coating of the macrophage cell membrane onto the surface of MTXNPs to generate biomimetic nanoparticles.

The purpose of this study was to examine whether the direct transplantation of extracted MMs onto nanoparticles would enable them to evade phagocytosis and effectively target AS *in vivo*. Hence, we conducted a comparison of the protein components present in total macrophage proteins, macrophage vesicles, MM/MTXNPs and MTXNPs using SDS–PAGE. As shown in Figure 3E, MTXNPs did not show protein bands, while MM-encapsulated MM/MTXNPs showed similar protein band profiles with total macrophage protein and macrophage vesicles, indicating that MM/MTXNPs successfully displaced MMs and retained their membrane proteins, which could enable MM/MTXNPs to perform macrophage-specific functions *in vivo* in subsequent experiments. VCAM-1 is a protein induced by cytokines expressed by the vascular endothelium that facilitates cell adhesion and is also recognized by integrin α4β1 [27]. The suppressor of cytokine signaling protein alpha (SIRPα) acts as an immune checkpoint protein that binds to the cell surface glycoprotein CD47 and thereby prevents the elimination of cells by the immune system [28, 29]. Macrophage surfaces contain various proteins; among them, we focused on those proteins that play a role in targeting and immune evasion. Therefore, we examined integrin α4β1 and CD47 protein components using western-blot analysis of macrophages, macrophage vesicles and MM/MTXNPs. Figure 3F demonstrates that MM/MTXNPs contained integrin α4β1 and CD47 proteins. This finding suggests that the MM/MTXNPs generated in this study possess the potential to achieve immune evasion and effectively target the lesion site of AS.

Given the GSH responsiveness of 3,3′-dithiodipropionic acid in HSP and the H_2_O_2_ responsiveness of PBAP, we aimed to investigate the responsive release of MTXNPs and MM/MTXNPs in this experiment. As observed from the overall trend of drug release (Figure 3G), the uncoated group (dotted line) exhibited a higher rate of drug release than the coated group (solid line). This signifies that the nanoparticles embedded within the cell membranes can slow down the drug-release process. Observations from each group indicated variations in the release rates in response to water, 1 mM H_2_O_2_, 1 mM GSH and 1 mM H_2_O_2_ + 1 mM GSH. Comparative analysis indicated that the release medium containing 1 mM H_2_O_2_ or 1 mM GSH exhibited a higher rate of MTX release than the aqueous solution group. Based on these findings, 1 mM H_2_O_2_+1 mM GSH exhibited the highest rate of drug release. The controlled release of drugs in the inflammatory environment of AS, aided by the unique ROS responsiveness, can potentially reduce drug toxicity and improve the effectiveness of AS treatment. Considering the above characteristics, the MM/MTXNPs synthesized in this study appear to hold great promise for the effective treatment of AS.

### Targeting and biosafety of biomimetic nanomaterials

**Figure 4.**
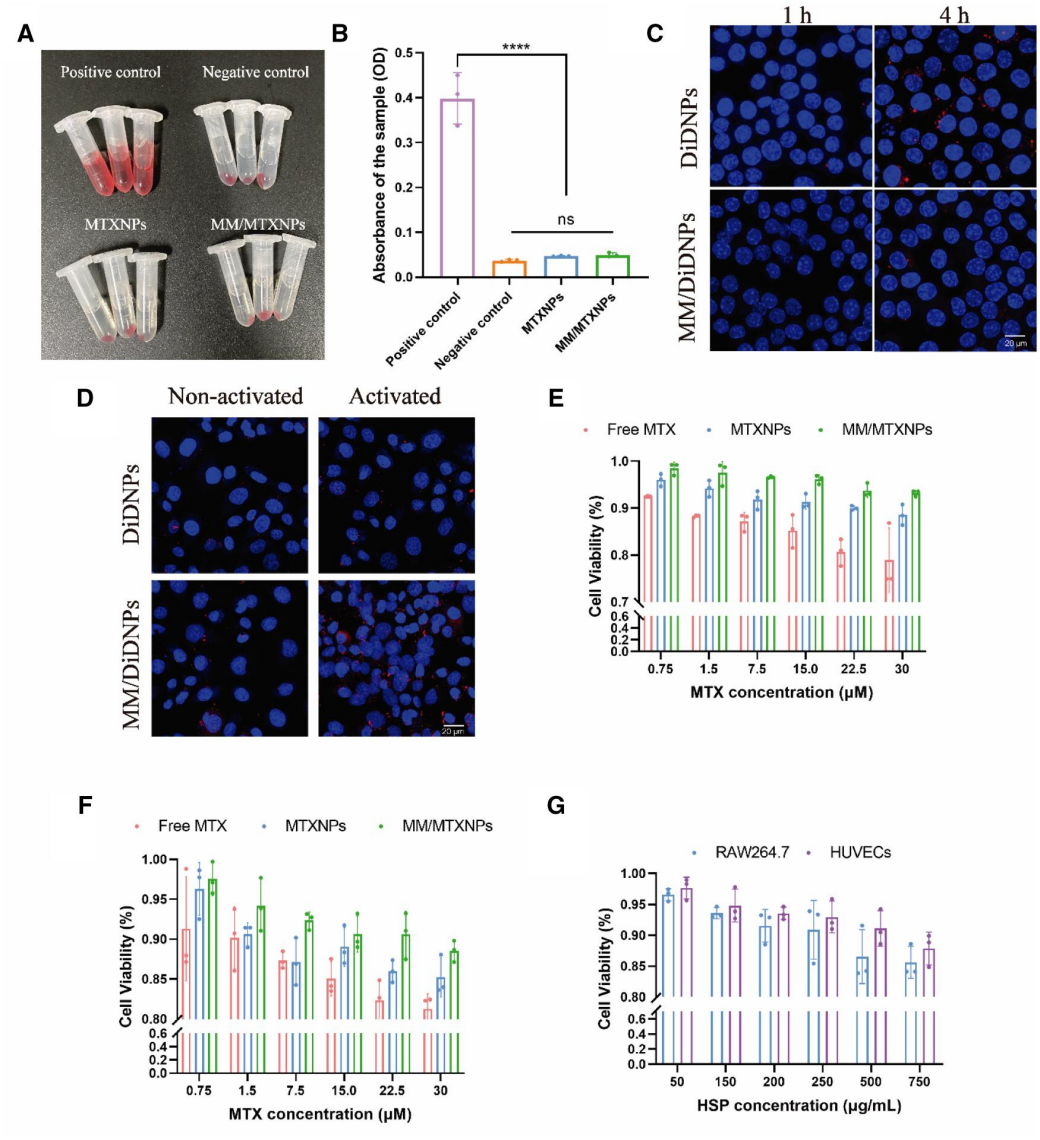
(**A**) Pictures of hemolysis tests including the positive control, negative control, MTXNPs and MM/MTXNPs. (**B**) Absorbance statistics of the hemolysis assay (*n* = 3). (**C**) Laser confocal images of uptake of DiDNPs and MM/DiDNPs by macrophages (DAPI-stained nuclei in blue, fluorescently labeled nanoparticles in red, the scale bar is 20 μm). (**D**) Laser confocal images of DiDNPs and MM/DiDNPs uptake by normal endothelial cells and inflammatory endothelial cells (the scale bar is 20 μm). (**E**) Cell viability of endothelial cells after treatment with MTX, MTXNPs and MM/MTXNPs (*n* = 3). (**F**) Cell viability of macrophages after treatment with MTX, MTXNPs and MM/MTXNPs (*n* = 3). (**G**) Cell viability of RAW264.7 cells and HUVECs after treatment with HSP (*n* = 3), ****P<0.0001; *ns*, no significance.

Materials or drugs used in living organisms must possess good biocompatibility. When materials are implanted or injected into the body, the erythrocyte membrane may rupture, leading to the dissolution of hemoglobin, which can result in significant harm to the body. The hemolysis test is an *in vitro* evaluation method that can directly measure the extent of damage to the material on red blood cells. Figure 4A depicts the centrifugation of blood after co-incubation with samples. The positive control group exhibited obvious hemolysis, whereas both MTXNPs, MM/MTXNPs and the negative control group exhibited deposition of red blood cells at the bottom of the centrifuge tube. The two experimental groups appeared light yellow, which was due to MTX being a yellow powder. In this experiment, the absorbance at 545 nm of each sample group was recorded in Figure 4B, and the resulting hemolysis rate was calculated (as shown in Supplementary Table S1). It was discovered that the hemolysis rate of MTXNPs was 2.7%, while that of MM/MTXNPs reached 3.6%, both of which complied with the national biosafety standard that requires biomedical materials to have a hemolysis rate below 5%. These results indicate that the MTXNPs and MM/MTXNPs synthesized in this study possess significant biological safety.

As specialized phagocytes of the immune system, macrophages possess the capability to detect and eliminate invading pathogens and apoptotic cells through phagocytosis. Theoretically, wrapping intact cell membranes directly onto the surface of nanoparticles could facilitate the transfer of various physiological functions of cell membranes, including recognition and signal transduction, as well as their ability to interact with the surrounding environment [30, 31]. The cell membrane of immune cells can be used as a coating for nanoparticles to evade the surveillance and clearance of the immune system through recognition by cells of the same type (homotypic recognition). In this study, macrophages were chosen to coat the surface of nanoparticles, which would allow them to escape phagocytosis by macrophages. With this objective in mind, we examined and compared the uptake of regular fluorescent nanoparticles by macrophages and fluorescent nanoparticles coated with macrophage cell membranes. Over time, the number of phagocytosed nanoparticles gradually increased. At 1 h of co-incubation with macrophages, DiDNPs started to cluster together, but there was no red fluorescence aggregation observed in nanoparticles coated with macrophages. After 4 h of co-incubation, DiDNPs had significantly aggregated in macrophages (Figure 4C). Interestingly, the accumulation of red fluorescence in cells treated with MM/DiDNPs was significantly decreased compared to that in cells treated with DiDNPs, indicating that macrophages could serve as a surface coating that could help nanoparticles evade macrophage phagocytosis. This study was based on the ability of VCAM-1, an adhesive protein expressed on endothelial cell membranes, to bind to integrin proteins on the surface of macrophage cell membranes. To verify this, we conducted an *in vitro* experiment whereby inflammatory endothelial cells and ordinary endothelial cells were co-incubated with DiDNPs and MM/DiDNPs, respectively. The results of this experiment are shown in Figure 4D. Observations of phagocytosis in inflammatory endothelial cells revealed that after 4 h of incubation with DiDNPs and MM/DiDNPs, only a small amount of red fluorescence was detected in the cells within the visual field. On the other hand, inflammatory endothelial cells co-incubated with MM/DiDNPs exhibited visible red fluorescent aggregation within the cells, suggesting that macrophage-coated nanoparticles can be specifically taken up by inflammatory endothelial cells by targeting Integrin-α4β1 on the surface of MMs and VCAM-1, which is highly expressed on the surface of inflammatory endothelial cells (Figure 4D, Supplementary Figure S1). Therefore, nanodrugs can be concentrated in the lesion site of AS.

To evaluate the potential toxicity of the synthesized nanoparticles, both endothelial cells and macrophages were separately seeded in 96-well plates. After co-incubation with the drugs for 8 h, cell viability was measured using the CCK-8 Kit. As demonstrated in Figure 4E (endothelial cells) and Figure 4F (macrophages), the cell activity gradually decreased as the concentration of MTX increased. However, even when the MTX concentration reached 30 μM, the cell survival rate in each group remained above 80%. Remarkably, MM/MTXNPs showed a higher cell survival rate than both MTX and MTXNPs. Subsequently, we investigated the cytotoxicity of the synthesized carrier HSP, and the results are presented in Figure 4G. Even at a concentration of 750 μg/ml, the cell survival rates of both endothelial cells and macrophages were still over 80%, suggesting that the nanoparticles synthesized in this study could reduce drug toxicity by potentially delaying drug release.

### Inhibition of lipid accumulation

**Figure 5.**
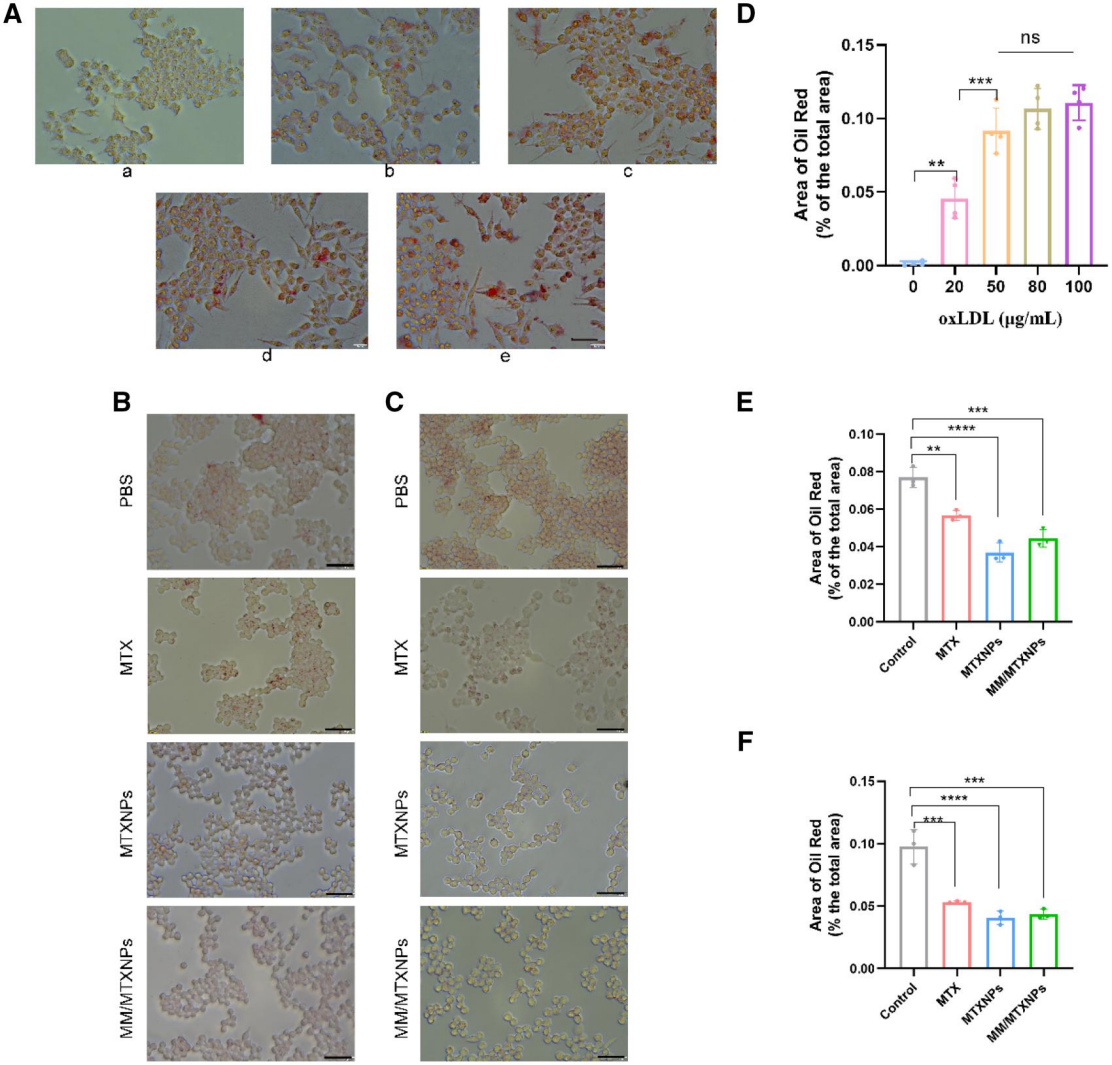
Inhibits lipid accumulation. (**A**) Stimulation of the creation of foam cells, oil red O staining of oxLDL after coincubation with macrophages, (a) 0 µg/ml oxLDL; (b) 20 µg/ml oxLDL; (c) 50 µg/ml oxLDL; (d) 80 µg/ml oxLDL; (e) 100 µg/ml oxLDL, the scale bar is 20 μm. (**B**) Oil red O staining of foam cells after coincubation with drugs; the scale bar is 20 μm. (**C**) Oil red O staining of macrophages after coincubation with oxLDL and drugs; the scale bar is 20 μm. (**D**) Statistical results of (A) (*n* = 3). (**E**) Statistical results of (B) (*n* = 3). (**F**) Statistical results of (C) (*n* = 3), **P<0.01, ***P<0.001, ****P<0.0001; *ns*, no significance.

During the development of AS lesions, low-density lipoprotein (LDL) accumulates in the subendothelial layer of the damaged vessel wall. After oxidative modification, LDL is taken up by macrophages, leading to the formation of foam cells [32]. This study aimed to determine the concentration of oxidized low-density lipoprotein (oxLDL) necessary to induce foam cell formation through co-incubation with monocyte-derived macrophages. Multiple concentrations of oxLDL were used to identify the most effective conditions for inducing foam cell formation. Oil red O staining was performed on macrophages co-incubated with various concentrations of oxidized oxLDL, and the resulting graphs are shown in Figure 5A. Quantitative analysis of oil red O staining for each group is provided in Figure 5B. The induction results for 80 or 100 μg/ml oxLDL were not significantly different from those for 50 μg/ml oxLDL. Therefore, to optimize the economic efficiency, a concentration of 50 μg/ml oxLDL was selected to stimulate foam cell formation in subsequent experiments.

Foam cell formation is a critical step in the development of atherosclerotic lesions, involving the transformation of macrophages through lipid accumulation [33]. To prevent excessive foam cell formation, reverse cholesterol transporters coordinate to export excess lipids from macrophages, thereby limiting their formation and slowing the progression of AS [34, 35]. Previous studies have demonstrated that MTX protects against ASCVD by promoting cholesterol transport in monocytes and macrophages and preventing the differentiation and activation of foam cells [23]. In the current study, macrophages were co-incubated with oxLDL to create a conducive environment for foam cell formation. Simultaneously, MTX, MTXNPs and MM/MTXNPs, each with an equal dosage of MTX, were co-incubated with cells and oxLDL for 12 h to evaluate intracellular lipid accumulation, as shown in Figure 5B and E. Both MM and MTXNPs significantly inhibited foam cell formation, indicating that MTX is effective in reducing lipid accumulation in macrophages and decreasing foam cell formation. The process of feeding macrophages oxLDL gradually induces their differentiation into foam cells, which can be reversed by reducing intracellular oxLDL levels to restore cellular homeostasis. This can be achieved through two distinct mechanisms: limiting oxLDL absorption and promoting oxLDL efflux [26]. Figure 5C and F demonstrate that treating foam cells with MTX, MTX nanoparticles (MTXNPs) or MM/MTXNPs at a consistent MTX dosage substantially reduces intracellular lipid content and partially reverses foam cell maturation. This provides evidence that MTX can effectively decrease lipid accumulation in macrophages and obstruct foam cell formation.

### *In vivo* targeting of atherosclerotic plaques

**Figure 6.**
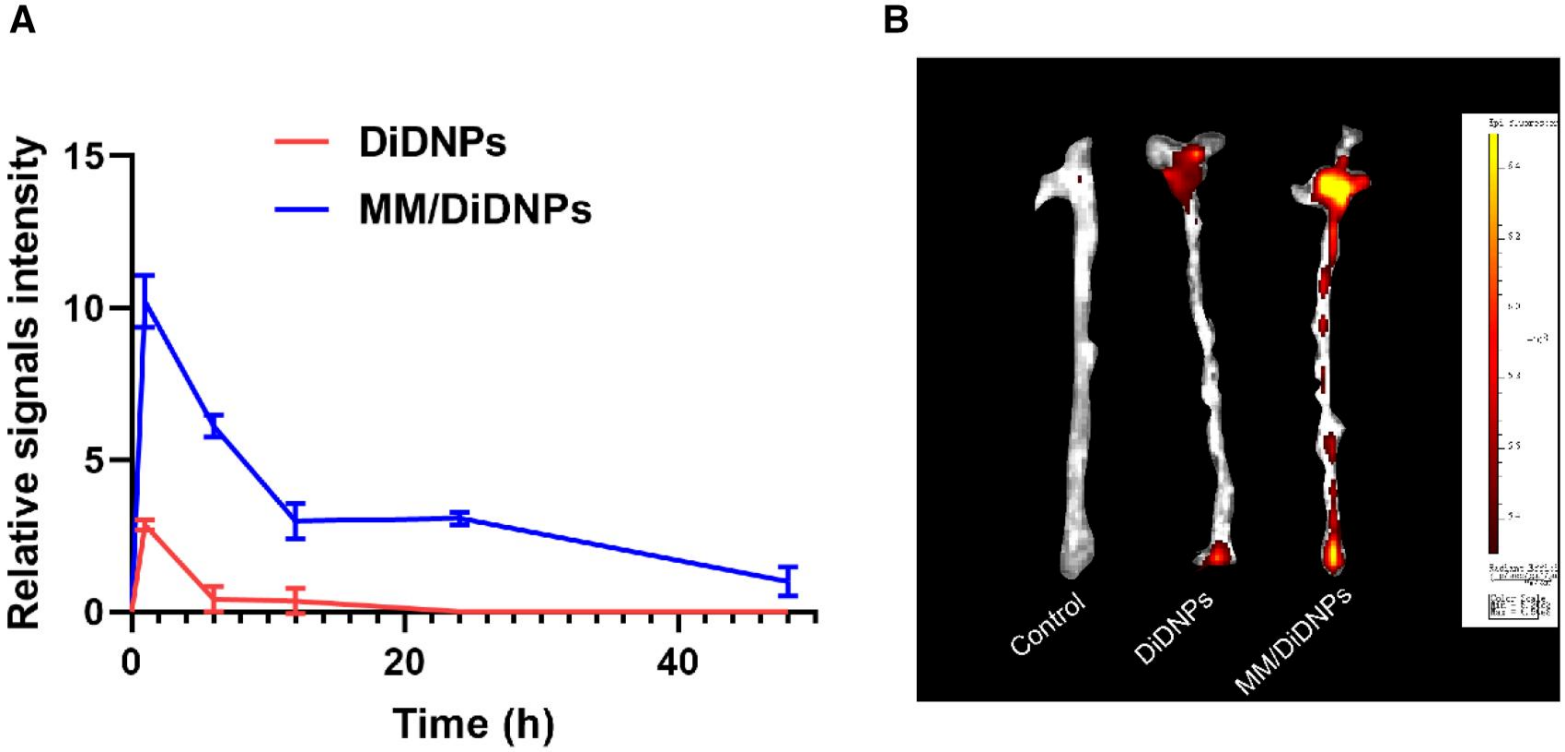
Targeting atherosclerotic plaques in ApoE^−/−^ mice. (**A**) Fluorescence intensity of DiDNPs and MM/DiDNPs circulating in mice at different times (*n* = 3). (**B**) Vascular fluorescence images of DiDNPs and MM/DiDNPs in mice after 24 h of circulation *in vivo*.

To investigate whether nanoparticles coated with macrophage cell membranes can evade phagocytosis *in vivo*, we conducted a study to assess the circulation of DiDNPs and MM/DiDNPs. We administered nanoparticles and collected blood samples from mice at specific time points to measure their fluorescence intensity. As shown in Figure 6A, MM/DiDNPs exhibited significantly prolonged circulation *in vivo* compared to DiDNPs. Following a 1-h circulation cycle, we observed an increase in fluorescence intensity for both DiDNPs and MM/DiDNPs. However, the fluorescence intensity of DiDNPs decreased significantly compared to MM/DiDNPs, suggesting that DiDNPs are primarily eliminated from mice within 1 h of injection. By 24-h postinjection, the fluorescence intensity of DiDNPs had reached 0, while MM/DiDNPs continued to exhibit fluorescence even after 48 h. These findings strongly indicate that nanoparticles coated with macrophage cell membranes have the capability of long-term circulation *in vivo*, presenting potential advantages for evading phagocytosis and enhancing nanoparticle stability.

To evaluate the ability of the nanoparticles to target plaques, we intravenously administered PBS, DiDNPs and MM/DiDNPs to ApoE^−/−^ mice via the tail vein. After a 24-h circulation period, the blood vessels were dissected and subjected to small animal imaging to assess nanoparticle fluorescence. Figure 6B depicts these observations. Our results revealed varying levels of fluorescence accumulation in the blood vessels for both the DiDNPs and MM/DiDNPs groups compared to the PBS control group. Furthermore, MM/DiDNPs exhibited significantly stronger fluorescence than DiDNPs, providing evidence that MM-coated fluorescent nanoparticles have the capability to target aggregation at plaque sites. In addition to targeting plaque sites, nanomedicine will also aggregate to a certain extent in the liver and kidney. This is because the liver and kidney are the main organs responsible for filtering blood and eliminating waste. They have a relatively high blood flow and possess the ability to engulf and process external substances (as shown in Supplementary Figure S1). These findings highlight the potential of these nanoparticles for targeted plaque imaging and therapy.

Endothelial dysfunction is a crucial event in the initiation and progression of AS. In this state, dysfunctional endothelial cells express adhesion molecules such as VCAM-1 and ICAM-1 and secrete chemokines to attract circulating mononuclear macrophages [36, 37]. Therefore, the MM-coated nanoparticles inherit the functional proteins of the macrophage cell membrane, allowing for the active targeting of dysfunctional endothelial cells. These nanoparticles exhibit prolonged circulation and the ability to specifically interact with these cells, facilitating effective accumulation in atherosclerotic lesions. Consequently, MM-coated nanoparticles hold great potential for targeted imaging and treatment of AS, offering promising opportunities in the field.

### *In vivo* therapeutic efficacy

To evaluate the therapeutic potential of macrophage cell membrane-coated nanoparticles in mice, we established an ApoE^−/−^ mouse model of AS and administered a drug via tail vein injection over 30 days, as demonstrated in Figure 6A. After the treatment period, the aorta of each mouse was stained with oil red O and imaged. The resulting micrographs of the blood vessels are presented in Figure 6B. As illustrated, Figure 6B shows substantial oil red O staining in the carotid artery, abdominal aorta and iliac artery of control mice injected with a PBS solution, indicating significant lipid deposition and atherosclerotic lesion formation in ApoE^−/−^ mice fed a high-fat diet. These findings provide valuable insights into the potential therapeutic effects of macrophage cell membrane-coated nanoparticles in the treatment of AS. However, the hydrophobic nature of MTX results in its rapid clearance in circulation, limiting its therapeutic efficacy. To overcome this issue, we modified the drug with a hydrophilic nanoshell, which moderately extended its circulation time in the body and demonstrated a certain level of therapeutic effect. Building upon this foundation, macrophage cell membrane-coated nanoparticles not only evade immune system clearance but also prolong circulation time within the body, thereby enhancing the likelihood of targeting atherosclerotic lesion sites. Additionally, *in vitro* studies have shown that a concentration of 150 U/ml hyaluronidase (HAase) effectively triggered the release of HA-based nanomaterials. Moreover, the atherosclerotic plaque microenvironment is known to exhibit high levels of hyaluronidase expression [38], further supporting the targeted delivery potential of these nanoparticles. It was suggested that the targeted delivery of HA-coated nanomaterials to atherosclerotic plaques, facilitated by the action of HAase, effectively promoted the rapid release of MTX, thereby significantly improving the therapeutic efficacy of this nanomedicine. As shown in Figure 6B, the MM/MTXNPs group exhibited the most favorable treatment response among all groups, demonstrating reduced lipid deposition in the aorta compared to all other treatment groups, demonstrating reduced lipid deposition in the aorta. Additionally, further analysis of the plaque area in the aorta (Figure 6C) revealed that in the control group, the plaque area accounted for 28.9% of the total aortic area. Treatment with MTX and MTXNPs resulted in an average plaque area of 24.9% and 22.0%, respectively, of the total aortic area. Notably, treatment with MM/MTXNPs significantly reduced the average plaque area to 12.3%, indicating robust inhibition of plaque formation and a significant therapeutic effect against AS in ApoE^−/−^ mice. In summary, the use of macrophage cell membrane-coated nanoparticles not only prolongs the circulation time of nanomedicine but also enhances targeted delivery to atherosclerotic plaque sites, thereby improving therapeutic efficacy and significantly inhibiting the development of AS plaques.

**Figure 7.**
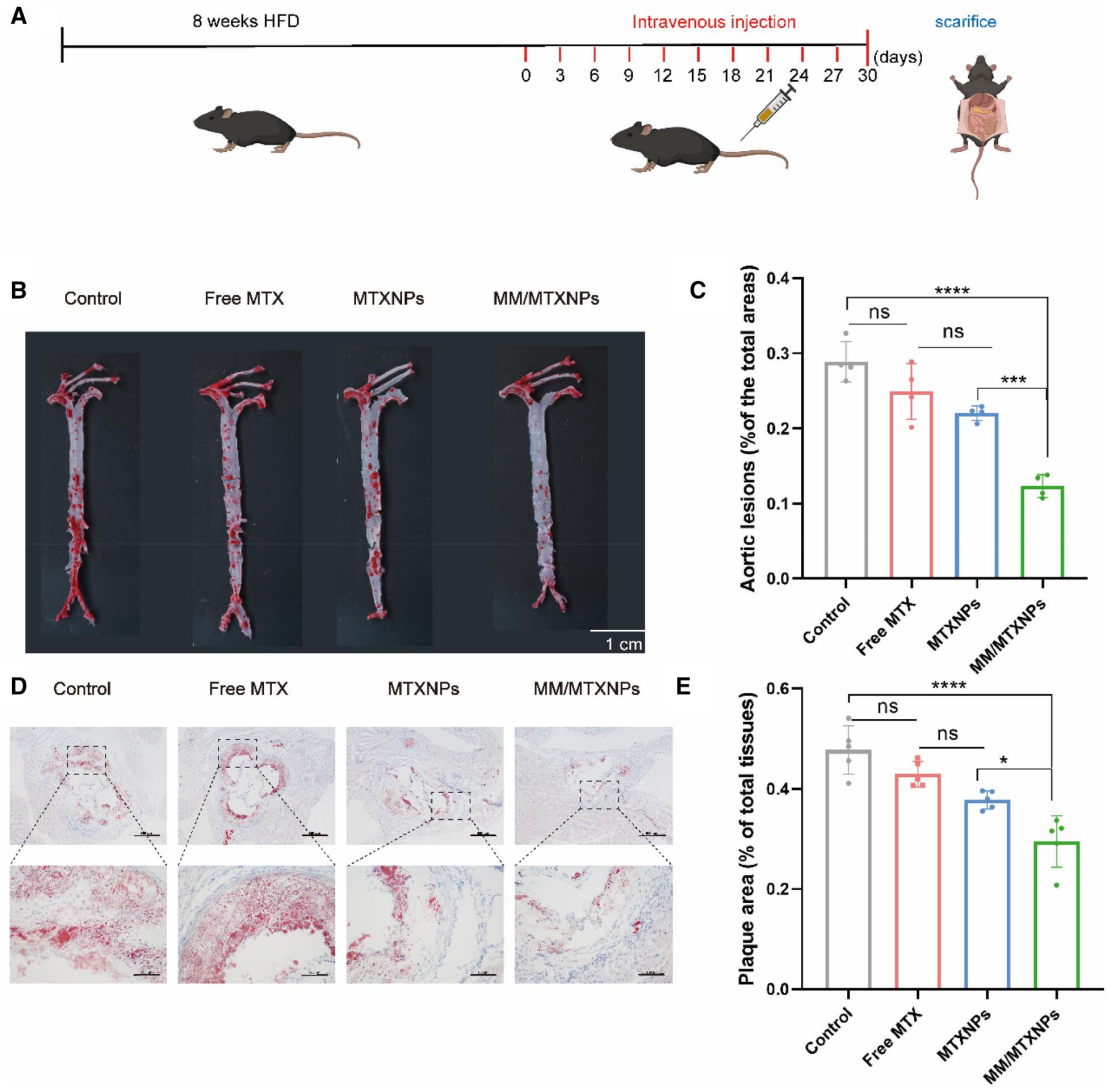
Therapeutic effects on atherosclerosis in ApoE^−/−^ mice. (**A**) ApoE^−/−^ mouse modeling and treatment model. (**B**) Images of oil red O staining of mouse aortas in different treatment groups. (**C**) Statistical results of plaque area as a percentage of the aortic area after different treatments (*n* = 5). (**D**) Oil red O staining of sections of the root of the aortic arch in mice after different treatments. The scale bar is 500 μm, and the enlarged image scale bar is 100 μm. (**E**) Statistical results of plaque area in the root of the aortic arch as a percentage of aortic area in mice after different treatments (*n *=* *5). *P <* *0.05, **P <* *0.01, ***P <* *0.001; *ns*, no significance.

Macroscopic staining and statistical analysis using Oil Red O revealed a significant amount of lipid deposition in the aorta of high-fat diet-fed ApoE^−/−^ mice, resulting in severe AS lesions. Additionally, evaluating lipid deposition within plaques is necessary to understand the severity of AS plaques. Accordingly, in this study, oil red O staining was performed on the root section of the mouse aortic arch, and the results are shown in Figure 7E. In Figure 7D, oil red O staining was used to visualize the root section of the mouse aortic arch after different treatments. The stained sections show that in ApoE^−/−^ mice injected with PBS, the area of the intravascular lumen was significantly reduced due to lipid deposition, and extensive lipid accumulation in the plaques led to severe AS lesions. Upon conducting additional statistical analysis, it was found that the plaque area at the root of the aortic arch in mice after undergoing different treatments (Figure 7E) varied. The results showed that the average plaque area in the control group injected with PBS accounted for 47.7% of the aortic area. In the MTX group, it was 42.9%, and in the MTXNPs group, it was 37.8%. The plaque proportion was significantly reduced to 29.4% in the MM/MTXNPs treatment group. This indicates that MM/MTXNPs treatment has proven to be highly effective in inhibiting plaque formation.

**Figure 8.**
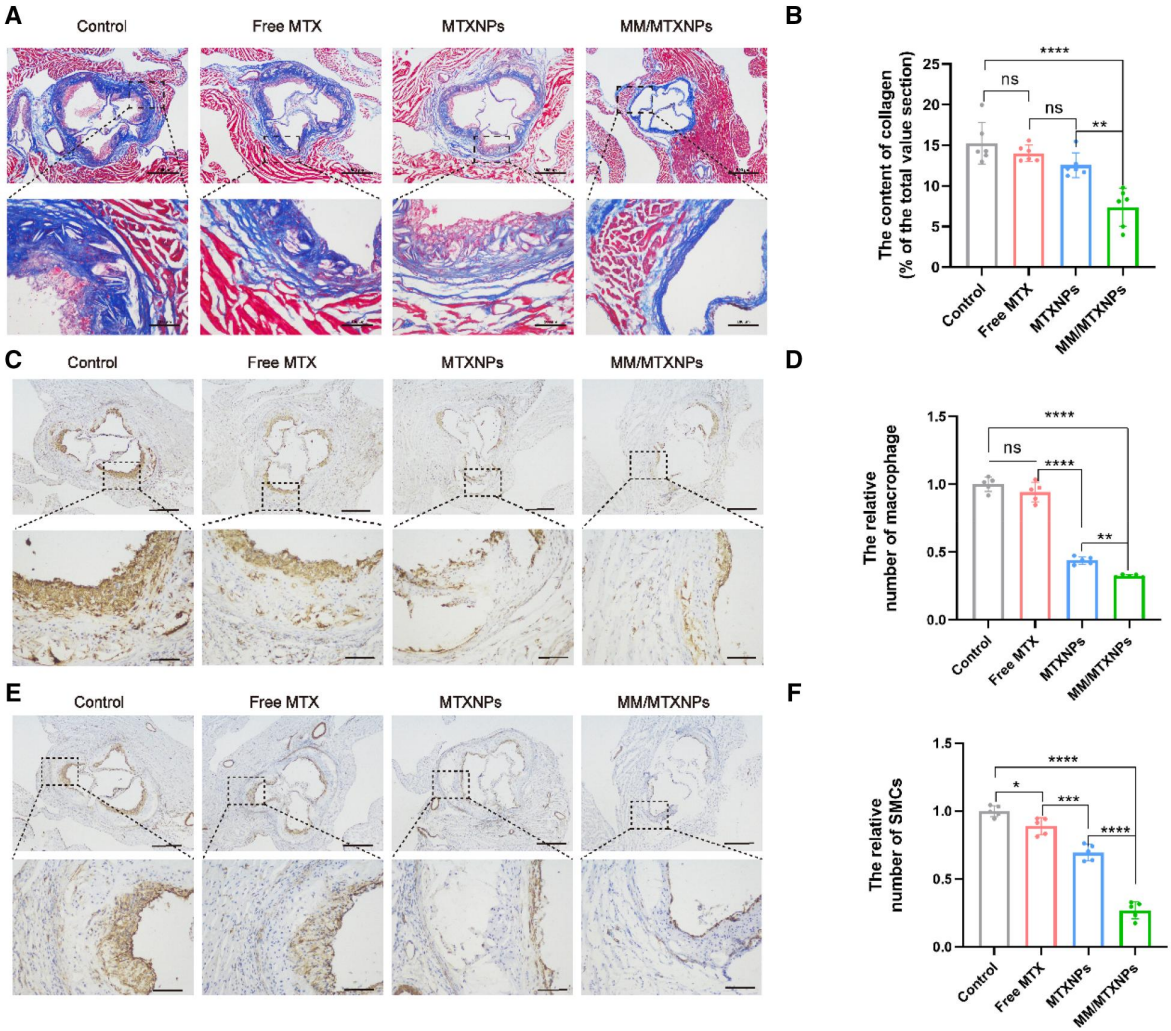
(**A**) Collagen staining of sections of the root of the aortic arch in mice after different treatments. (**B**) Statistical results of collagen area as a percentage of plaque area in root sections of the aortic arch of mice after different treatments (*n* = 5). (**C**) Immunohistochemistry of CD68 in sections of the root of the aortic arch of mice after different treatments. (**D**) Relative macrophage counts in root sections of the aortic arch of mice after different treatments (*n* = 5). (**E**) Immunohistochemistry of α-SMA in sections of the root of the aortic arch of mice after different treatments. (**F**) Relative smooth muscle cell counts in root sections of the aortic arch in mice after different treatments (*n* = 5). The scale bar is 500 μm and the enlarged image scale bar is 100 μm. *P <* *0.05, **P <* *0.01, ***P <* *0.001; ns, no significance.

Smooth muscle cells are responsible for producing new extracellular matrix in AS lesions, which is primarily composed of collagen. The abnormal deposition and metabolism of collagen play a critical role in the formation and development of vulnerable AS plaques [39]. This study detected collagen deposition in the root section of a mouse aortic arch after different treatments using collagen staining. After MTX and MTXNPs treatment, a slight reduction was observed in the collagen content (blue in Figure 8A), while a significant reduction was observed after MM/MTXNPs treatment. Upon further data analysis (Figure 8B), it was concluded that the control group of mice injected with PBS had an average collagen content of 15.28% in the root sections of the aortic arch. The collagen area accounted for 14.02% of the plaque area in the MTX treatment group and 12.56% in the MTXNPs treatment group. Compared to the control group, the therapeutic effect was considered weak, with an average collagen area accounting for only 7.39% of the plaque area in the MM/MTXNPs treatment group. However, the collagen content in the MM/MTXNPs treatment group showed a significant reduction compared to the three groups injected with PBS, MTX and MTXNPs, indicating a considerable therapeutic effect.

During the early stages of AS, macrophages are attracted to accumulate in the subendothelial layer or neointima. This attraction is attributed to the expression of endothelial adhesion molecules and the presence of subendothelial lipoproteins [40]. Macrophages take up lipoproteins, leading to the formation of foam cells. The continued accumulation of foam cells promotes the storage of plaque lipids and further contributes to plaque growth, thus fostering the development of AS [10]. Thus, understanding the macrophage content in AS plaques can help evaluate the extent of AS lesions. Inhibiting macrophage proliferation can also lead to a reduction in the lesion area. CD68 is a specific protein marker for macrophages. Immunohistochemical staining of CD68 in the root section of the mouse aortic arch can indicate the therapeutic impact of MM/MTXNPs. The plaques in the control group exhibited a significant presence of macrophage markers, with a slight decrease observed after one month of MTX treatment. Nevertheless, treatment with MTXNPs and MM/MTXNPs resulted in a notable reduction in macrophage content at the lesion site (Figure 8C). After one month of treatment, further data analysis revealed that the macrophage content was 94.3% in the MTX injection group, 43.9% in the MTXNPs group and 23.4% in the MM/MTXNPs group (Figure 8D). This indicates that, compared to other treatment groups, the MM/MTXNPs group effectively reduces the number of macrophages at the lesion site and exhibits the most significant therapeutic impact.

Vascular smooth muscle cells are essential for maintaining the proper function of the vascular system. They form the body of most blood vessels and assist in the contraction and regulation of blood flow. During the initial stages of AS, specifically fiber cap formation, SMCs migrate to and proliferate in the intima, leading to vascular wall thickening and lumen narrowing, which contributes to the pathological progression of AS. SMCs play a crucial role in the development, advancement and stability of plaques through a process known as phenotypic transformation, involving their proliferation, dedifferentiation and migration. The trans-differentiation of SMCs into ‘inflammatory’ macrophage-like cells may promote the instability of AS lesions by initiating an inflammatory response [41]. The control group mice exhibited a significant number of smooth muscle cells within the plaques in the aortic arch root section. However, treatment with MTX and MTXNPs resulted in a notable reduction in smooth muscle cells within the plaques. In contrast, the therapeutic effect of MM/MTXNPs was reduced (Figure 8E). According to further analysis (Figure 8F), treatment with MTX and MTXNPs decreased the number of smooth muscle cells in the AS plaques of the mouse aortic arch to 81.3% and 63.2% of the untreated group, respectively, indicating a significant decrease. On the other hand, the number of smooth muscle cells in the MM/MTXNPs treatment group was only 17.6% of that in the control group. This indicates that MM/MTXNPs efficiently inhibit the proliferation of smooth muscle cells in AS plaques compared to MTX and MTXNPs.

In conclusion, treatment with MM/MTXNPs resulted in a significant reduction in the number of collagen, macrophages and smooth muscle cells at the plaque site in AS mice. Specifically, the amount of collagen decreased by 5.89%, macrophages decreased by 76.6% and smooth muscle cells decreased by 82.4%.

### Biosafety assessment

**Figure 9.**
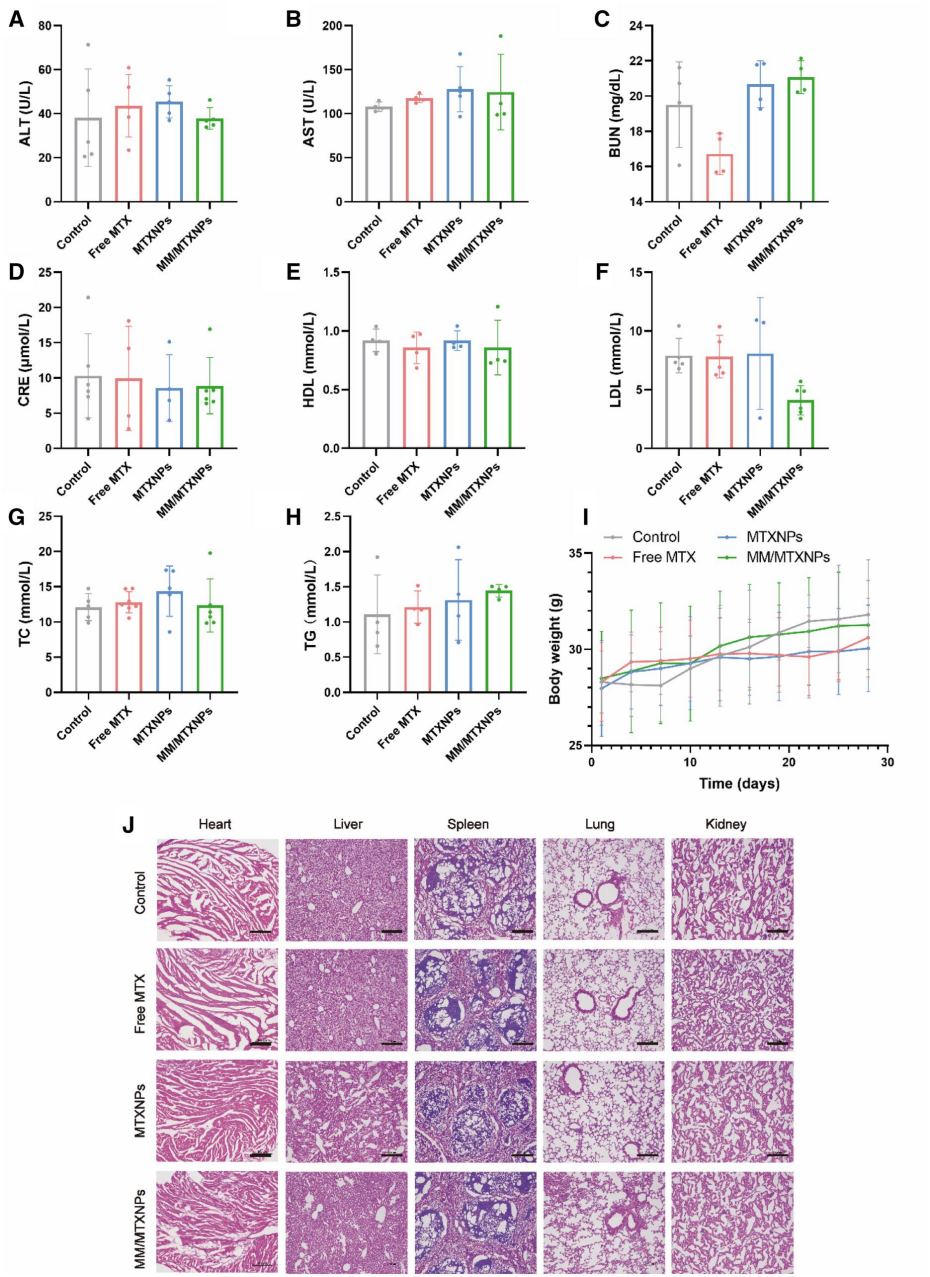
Biosafety assessment. (**A–D**) Blood biochemical indexes (*n* = 5) ALT: 10.06–96.47 U/l; AST: 36.31–235.48 U/l; BUN: 10.81–34.74 mg/dl; CRE: 10.91–85.09 μmol/l; HDL: 1.28–2.65 mmol/l; LDL: 0.12–0.26 mmol/l; TC: 2.05–4.16 mmol/l; TG: 0.84–2.72 mmol/l. (**E–H**) Four items of blood lipid tests (*n* = 5). (**I**) Changes in the body weight of mice during treatment (*n* = 5). (**J**) HE staining (the normal ranges are as follows: ALT: 10.06–96.47 U/l; AST: 36.31–235.48 U/l; BUN: 10.81–34.74 mg/dl; CRE: 10.91–85.09 μmol/l; HDL: 1.28–2.65 mmol/l; LDL: 0.12–0.26 mmol/l; TC: 2.05–4.16 mmol/l; TG: 0.84–2.72 mmol/l).

After one month of treatment, the analysis of blood composition in each group of mice showed similar and normal levels of red blood cells (RBCs) (Supplementary Figure S2A), white blood cells (WBCs) (Supplementary Figure S2B) and hemoglobin (HGB) (Supplementary Figure S2D). The platelet count (PLT) (Supplementary Figure S3C) in the MTXNP treatment group was significantly lower than that in the control group, which could be attributed to MTXNP-induced anemia. However, the PLT index in mice from the MM/MTXNPs group was within the normal range, suggesting that MM/MTXNPs exhibited good blood safety. The computer analysis of mouse serum (Figure 9A–D) indicated no significant differences in the biochemical indexes between the control group and all treatment groups. This indicates that the nanoparticles developed in this study did not exhibit any toxicity to liver and kidney tissue.

Lipid detection is used to assess the presence of dyslipidemia, including total cholesterol (TC), triglyceride (TG), high-density lipoprotein cholesterol (HDL) and low-density lipoprotein cholesterol (LDL) levels. The test results depicted in the figure indicated no significant differences in HDL (Figure 9E), TC (Figure 9G) and TG (Figure 9H) levels between the untreated group and the treatment groups. However, the LDL (Figure 9F) level in the MM/MTXNPs group was significantly lower than that in the control group and other treatment groups, showing a reduction in LDL levels. It is important to note that abnormal LDL levels are associated with an increased risk of cardiovascular and cerebrovascular diseases [42]. Lowering LDL levels is a crucial target for managing blood lipids, particularly for patients with such diseases. These findings suggest that MM/MTXNPs effectively reduce ‘bad’ cholesterol levels in ApoE^−/−^ mice, which is beneficial for reducing the occurrence of AS diseases. Further research and investigation are warranted to explore the potential implications of these results.

To assess the safety of the nanomaterials, H&E staining was performed on slices of mouse heart, liver, spleen, lung and kidney to evaluate the overall health of these vital organs after treatment. One month after caudal venous injection, the HE staining results (Figure 9J) demonstrated that none of the three treatment groups (MTX, MTXNPs and MM/MTXNPs), as well as the placebo group injected with PBS, exhibited any organic lesions. Specifically, the heart tissue showed clear and intact nuclear distribution, a complete shape and neatly arranged myocardial fibers. The liver cells and lobules displayed intact morphology, indicating healthy tissue. Additional evaluation and description of the other organs (spleen, lung and kidney) are necessary to provide a comprehensive understanding of their health status. No diffusion of the splenic pulp was observed, indicating intact splenic tissue, with a clear boundary between the red pulp and white pulp. The alveoli in the lungs were evenly distributed and neatly arranged, contributing to normal lung function. The renal tubular epithelial cells maintained their normal morphology, and no hyperplasia was observed in the glomeruli, suggesting proper kidney function. Overall, after one month of drug administration, the major organs of the mice maintained their proper morphology, indicating favorable biosafety of the nanoparticles developed in this study. Furthermore, analyzing the weight records of mice (Figure 9I) during the treatment period revealed a consistent upward trend, indicating that the administration of the medication had no impact on the appetite of the mice.

## Discussion

HA, which has high biosafety, was connected to 3,3’-dithiopropionic acid and PBAP groups with ROS responsiveness, generating a ROS-responsive amphiphilic carrier called HSP. MTX, known for its ability to inhibit lipid accumulation, was encapsulated within HSP using a nanoprecipitation method to form MTXNPs. The macrophage cell membrane was used to construct the shell of the MTXNPs, providing the nanoparticles with a ‘stealth’ functionality and the capability to target atherosclerotic plaques. MM/MTXNPs exhibited a ‘core-shell’ structure and retained characteristic macrophage surface proteins, including integrin α4β1 and CD47. This enables immune evasion and targeted delivery to atherosclerotic lesions. MTXNPs exhibited characteristics of responsive drug release due to the ROS-responsive nature of HSP, while the presence of MM enabled sustained drug release, providing MM/MTXNPs with advantages in drug delivery. Further research and experimentation are warranted to explore the full potential of these drug delivery systems.

In various *in vitro* experiments aimed at understanding the transport of oxLDL, MTX demonstrated the ability to regulate its transport. In comparison to MTXNPs and free MTX, MM/MTXNPs exhibited a stronger impact on reducing oxLDL accumulation in macrophages during *in vivo* experiments. This enhanced effect can be attributed to the immunoevasive and targeted capabilities of the MM-coated surface of MM/MTXNPs, as confirmed by *in vitro* confocal fluorescence microscopy. Furthermore, small animal imaging validated the targeted nature of the nanoparticles, with mice injected with MM/DiDNPs displaying higher fluorescence signals at the site of AS lesions in blood vessels. After one month of drug treatment via tail vein injection in ApoE^−/−^ mice, the MM/MTXNPs group showed a significant reduction in plaque area within the aorta. Moreover, MM/MTXNPs were effective in reducing lipid deposition, decreasing the levels of collagen, MMP-9, macrophages and smooth muscle cells within the plaque, and effectively inhibiting the progression of AS. These findings demonstrate the robust potential of the nanoparticles developed in this study for suppressing the progression of AS *in vivo*.

In conclusion, the findings of this study support the effectiveness of using ROS-responsive MTX nanoparticles coated with MMs for targeted drug delivery to AS plaque sites. The nanoparticles have shown the ability to induce responsive drug release, which contributes to their beneficial effect on the prevention and progression of AS. Further research and clinical studies are needed to fully explore the therapeutic potential of these innovative nanocarriers.

## Supplementary Material

rbae102_Supplementary_Data

## Data Availability

All date generated or analyzed during this study are included in this published article.
