# Supplementary material for: Biomimetic ROS-responsive hyaluronic acid nanoparticles loaded with methotrexate for targeted anti-atherosclerosis
Source: Regen Biomater. 2024 Aug 20;11:rbae102. doi: 10.1093/rb/rbae102 (PMC11474234; doi:10.1093/rb/rbae102)
Supplement: rbae102_Supplementary_Data [file rbae102_supplementary_data.docx]

**Supporting Information**

**Biomimetic ROS-responsive hyaluronic acid nanoparticles loaded with methotrexate for targeted anti-atherosclerosis**

Bingyi Li^1#^, Mei He^4#^, Zichen Xu^2^, Qianting Zhang^2^, Liyuan Zhang^1,2^, Shuang Zhao^2^, Yu Cao^2^, Nianlian Mou^2^, Yi Wang^3^*, Guixue Wang^1,2^*

1. JinFeng Laboratory, Chongqing 401329, China
2. Key Laboratory for Biorheological Science and Technology of Ministry of Education, State and Local Joint Engineering Laboratory for Vascular Implants, Bioengineering College of Chongqing University, Chongqing 400030, China
3. College of Basic Medical Sciences, Chongqing Medical University, Chongqing 400016, China
4. Chongqing University Cancer Hospital, Chongqing 400030, China

*** Corresponding authors: wanggx@cqu.edu.cn (G Wang); wwewwy@163.com (Y Wang)**

**# Co-first author**

**Table S1 Hemolysis rates of MTXNPs and MM/MTXNPs (*n* = 3)**

| Sample | The absorbance of the sample | | | Average | Hemolysis rate（%） |
| --- | --- | --- | --- | --- | --- |
|  | 1 | 2 | 3 |  |  |
| Positive control | 0.337 | 0.45 | 0.408 | 0.398 |  |
| Negative control | 0.039 | 0.038 | 0.034 | 0.037 |  |
| MTXNPs | 0.048 | 0.047 | 0.046 | 0.047 | 2.7 |
| MM/MTXNPs | 0.048 | 0.055 | 0.046 | 0.050 | 3.6 |


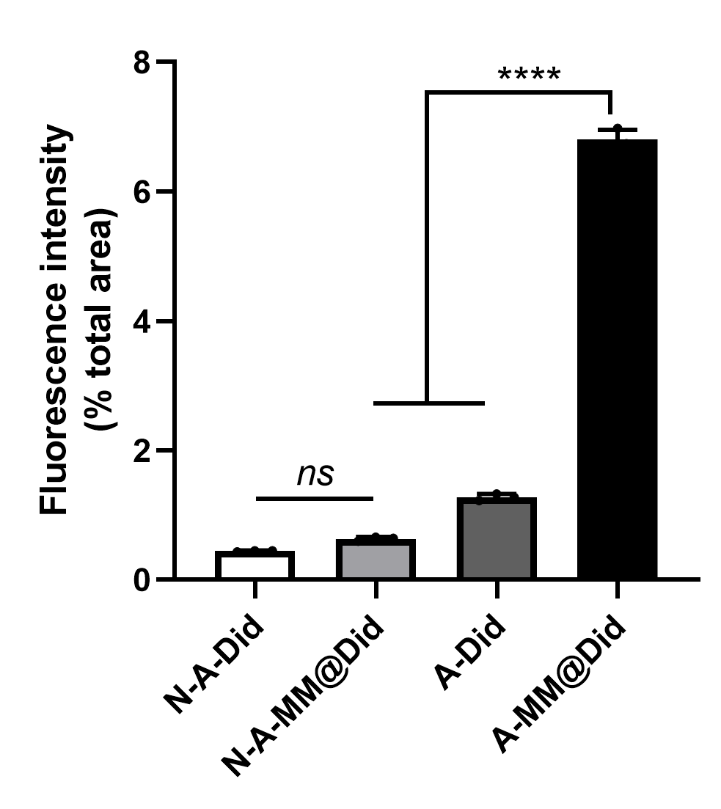


Figure S1 Statistical results of laser confocal imaging of normal endothelial cells and inflammatory endothelial cells uptake of DiDNPs and MM/DiDNPs (n=3). N-A-Did: DiDNPs uptake by Non-Actived endothelial cells, N-A-MM@Did: MM@DiDNPs uptake by Non-Actived endothelial cells, A-Did: DiDNPs uptake by Actived endothelial cells, A-MM@Did: MM@DiDNPs uptake by Actived endothelial cells. *****p*＜0.0001; *ns*, no significance.


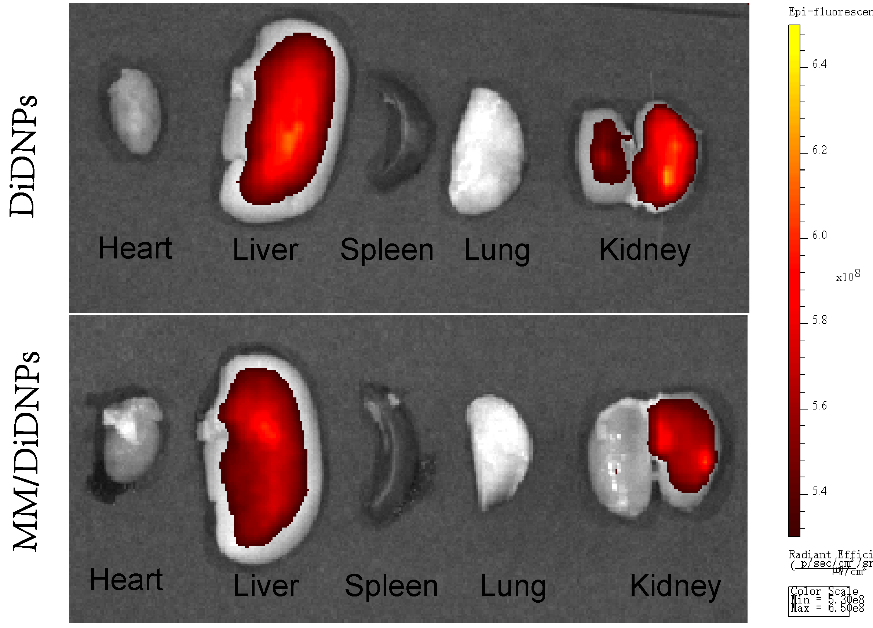


Figure S2 Ex vivo fluorescent images of DiDNPs and MM/DiDNPs in the major

organs at 24 h after i.v. injection (n = 3)


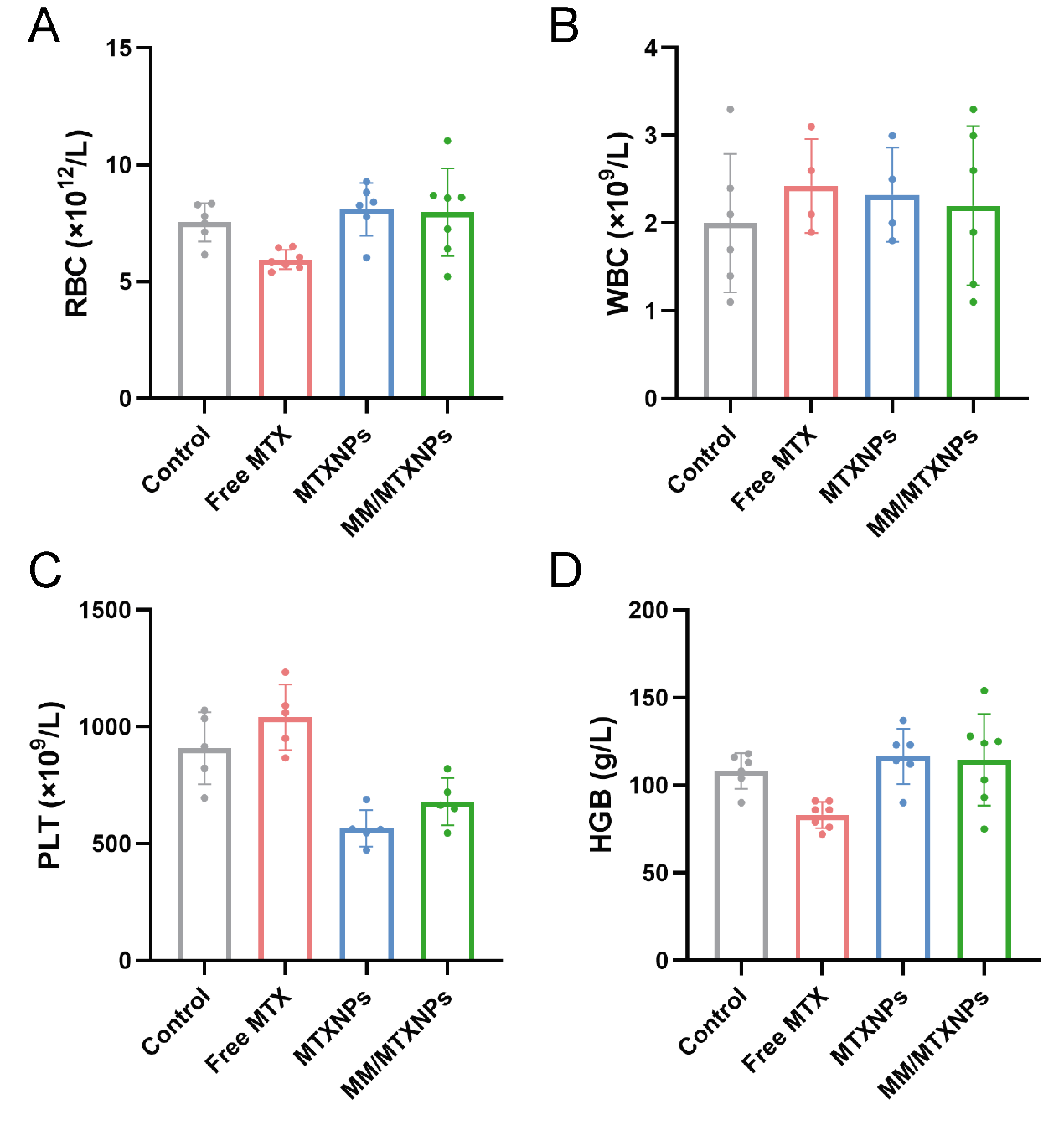


Figure S3 Biosafety assessment. (A-D) Blood routine test (n = 5).
